# Exudate identification in retinal fundus images using precise textural verifications

**DOI:** 10.1038/s41598-023-29916-y

**Published:** 2023-02-17

**Authors:** Maryam Monemian, Hossein Rabbani

**Affiliations:** Medical Image and Signal Processing Research Center, Isfahan University of Medical Sciences, Isfahan, Iran

**Keywords:** Biomedical engineering, Imaging techniques, Image processing

## Abstract

One of the most salient diseases of retina is Diabetic Retinopathy (DR) which may lead to irreparable damages to eye vision in the advanced phases. A large number of the people infected with diabetes experience DR. The early identification of DR signs facilitates the treatment process and prevents from blindness. Hard Exudates (HE) are bright lesions appeared in retinal fundus images of DR patients. Thus, the detection of HEs is an important task preventing the progress of DR. However, the detection of HEs is a challenging process due to their different appearance features. In this paper, an automatic method for the identification of HEs with various sizes and shapes is proposed. The method works based on a pixel-wise approach. It considers several semi-circular regions around each pixel. For each semi-circular region, the intensity changes around several directions and non-necessarily equal radiuses are computed. All pixels for which several semi-circular regions include considerable intensity changes are considered as the pixels located in HEs. In order to reduce false positives, an optic disc localization method is proposed in the post-processing phase. The performance of the proposed method has been evaluated on DIARETDB0 and DIARETDB1 datasets. The experimental results confirm the improved performance of the suggested method in term of accuracy.

## Introduction

Retina is an important body organ responsible for human vision. Diabetic Retinopathy (DR) is a salient disease which infects the people with the long history of diabetes. DR is developed as a result of high blood sugar levels that damages the retina. In fact, in DR the blood vessels which are responsible for nourishing the retina are damaged. It leads to the fluid and blood leakage to retina. If DR is not identified in an appropriate time, it may cause irreparable damages to eye vision^1^. A high percent of peoples with 10 years history of diabetes are infected with DR. Early identification and treatment of DR can control the disease and prevent from harmful damages to eye vision^1,2^.

Before the patients influenced by DR feel problem in their vision, DR may be in progress. Therefore, performing periodic eye exams in periodic time intervals is very important. One imaging modality which is beneficial in capturing from retina is fundus^3–5^. The demonstrations of DR in the fundus images include red-lesions such as Hemorrhages and Micro-aneurysms and also bright lesions like soft and hard exudates (HE)^1^. Manual verification of such images is a tedious, time-consuming process which may not be free of error. Therefore, to propose novel automatic methods for accurate evaluation of such images is of high importance^6^.

With no doubt, HEs are from the most prevalent signs of DR in the early stages. The leakage of blood from retinal vessels can form HEs which are characterized by bright lesions with sharp margins^1^. A number of research works were proposed for identifying HEs in retinal fundus images^7–31^. However, the variety of size and shape makes the process of HE identification challenging.

In this paper, an automatic method for the diagnosis of HEs is proposed. This method consists of three phases which are pre-processing, semi-circular regions verification and post-processing. In the first phase, the quality of image is improved to be prepared for processing operations. In the second phase, a pixel-wise approach is suggested where around each pixel several semi-circular regions are considered. For each semi-circular region, the intensity changes along several directions and different distances are verified. If it is possible to find several consecutive semi-circular regions around each pixel which contain sufficient intensity changes, the related region is considered as a part of HE. Since the method allows to consider intensity change in different distances from the initial pixel, the selected region is not necessarily a homogeneous region with a specific shape. In other words, it can be of various shapes and thanks to this point, the method is capable of identifying HEs with different shapes. In the third phase, the optic disc is localized to reduce the number of false positives.

The main contributions of this paper are summarized as follows.

- To find the HEs without the need to segmenting blood vessels or optic disc.
- To propose a procedure for detecting HEs with different shapes.
- To focus on finding intensity change in different directions and also distances.
- To provide a freedom degree for choosing the hard exudates. In fact, the pixels for which a number of neighbor regions satisfy the conditions of entrance area to an HE are considered as parts of HEs. In fact, it is not necessary for all the neighbor regions around a pixel to be an entrance area to an HE.

The rest of paper is organized as follows. Section "Related works" includes the explanations of existing works in the field of identification of HEs. The proposed method is presented in Sect. "Method". Section "Experimental results" contains the experimental results. Finally, the concluding remarks are presented in Sect. "Discussion".

## Related works

In this section, the most important works in the field of HE detection in retinal fundus images are explained. In^8^ a method for extracting HEs is proposed which contains pre-processing, candidate extraction, feature extraction, classification and post-processing phases. At first, contrast and luminosity improvement algorithms are applied in the first phase. Next, the image color space is transformed to CIELAB color space with the help of Super-pixel Linear Iterative Clustering (SLIC) method. Then, a set of features including a contextual feature and multi-channel features are extracted for the candidates. In the post-processing phase, the optic disc is localized in the image to exclude the mistakenly chosen regions for HE.

In^10^ a method for detecting healthy and pathological retinal regions including red and bright lesions is proposed. This method works based on the analysis of Local Binary Pattern (LBP) texture descriptor and morphological operations. In^11^ an automatic method for the segmentation of exudates is proposed. This method needs the initial segmentation of retinal vessels and also introduces new approaches for detecting reflections and artifacts in the pre-processing stage. Then, morphological features are utilized for finding the exudate candidates and random forests are used for classifying the candidates.

In order to classify the different stages of DR, a detection and segmentation approach for blood vessels, optic disc, hemorrhages, micro-aneurysms, and exudates is proposed in^12^. For segmenting exudates, firstly optic disc is localized through intensity variation algorithm and removed. Then, an inverse surface adaptive thresholding is used for finding exudates. In^13^ a method for detecting red and bright lesions is proposed which firstly improves the image quality and estimates background, vessel structure, fovea and optic disc. Then, the image is decomposed into several layers and the distinguishing features of red and bright lesions are extracted from the obtained layers. Then, Multi-Layer Perceptron (MLP) is used to classify true candidates from false ones. Automated detectors for hemorrhages, micro-aneurysms and bright lesions are proposed in^14^. For detecting red-lesions, three points are considered. In the first point, a low level wavelet is used for finding small dot bright lesions. Then, high level wavelet and texture and cartoon decomposition are utilized for finding medium, large ones.

A method for automatic detection of red and bright lesions in fundus images is suggested in^16^. After pre-processing operations including blood vessel and optic disc removal, a band-pass filter is designed for enhancing the contrast between bright lesions and background. In order to determine candidate bright lesions, matched filtering and Laplacian of Gaussian filtering are utilized.

In^20^ several deep learning methods for the purpose of exudate detection are compared. Different classifiers and various layers are used to maximize the values of sensitivity and specificity.

To the best of our knowledge, this is the first work focusing on the identification of exudates with different shapes with simple computations. In fact, no research work has identified shapes with different appearances without blood vessel segmentation, artefact removal and illumination correction. The proposed method relies on finding intensity changes in all directions and non-necessarily equal distances from each pixel.

## Method

In this section, the proposed method called Hard Exudate Detection with Semi-circular Region Analysis (HED-SRA) is explained in details. As mentioned before, HED-SRA includes three main phases which are pre-processing, semi-circular region verification and post-processing. All phases are detailed in the following.

### Pre-processing

Since the green channel of retinal fundus image has the highest contrast, firstly the green channel of image is considered for processing operations. This point is similar to what has been considered in^32–36^. Also, normalization process is applied on the image.

### Semi-circular region verification

In this phase, the main operations for the determination of HEs are performed.

**Figure 1:**
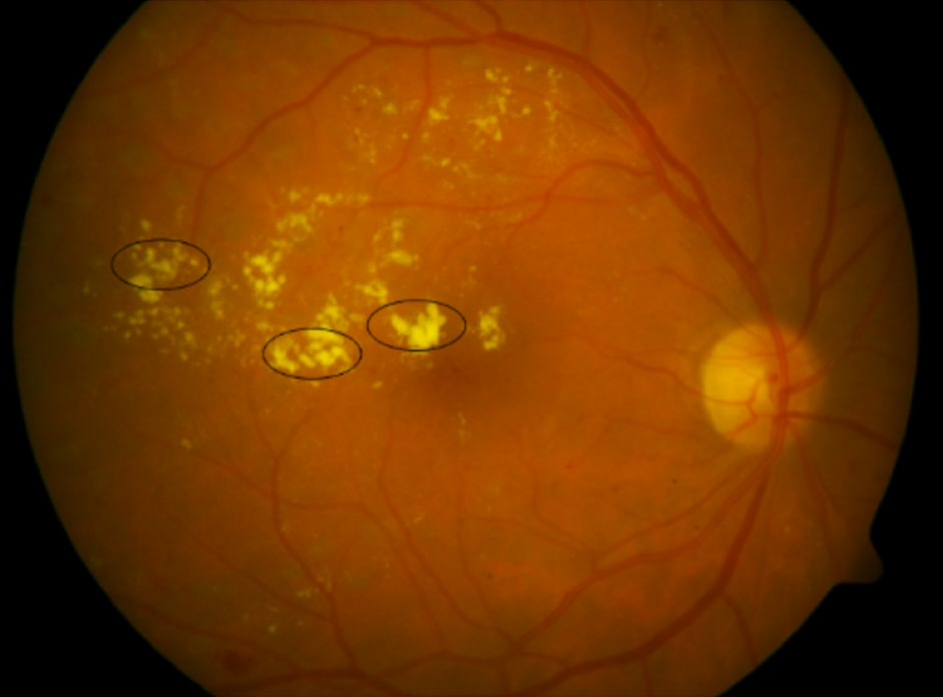
A fundus image including HEs.

Firstly, the appearance features of HEs should be considered. It should be noted that HEs are light or yellowish regions in the retinal fundus images. Figure 1 presents a fundus image including HEs shown by black ellipses.

**Figure 2:**
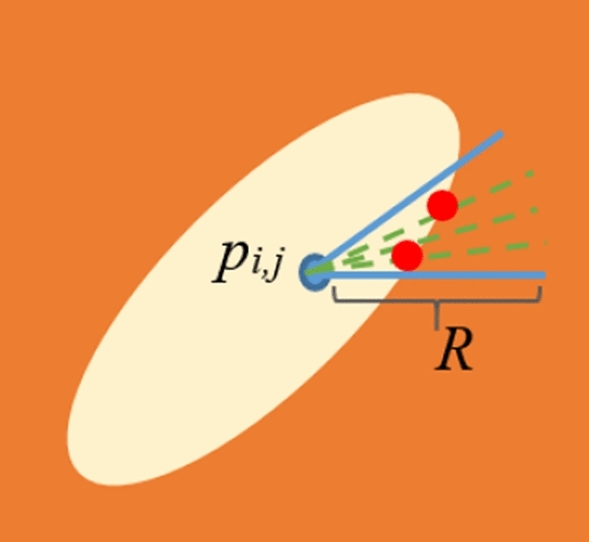
A schematic view for a semi-circular region around *p*_*i,j*_.

Firstly, all pixels are separately considered. Around each pixel, several semi-circular regions are considered. By “semi-circular region”, we mean a part of circle with certain radius centered at the pixel of interest. This part should cover a range of angles that is a subset of [0, 2π]. The radius of such a circle is denoted by *R*. For simplicity, we consider 8 non-overlapping semi-circular regions in a whole circle. Let *SC*_*i*_ denote the *i*th semi-circular region. The range of angles covered by *SC*_*i*_ is denoted by ${\theta}_{i}$ and is equal to [$(i-1)\frac{\pi }{4}$,$i\frac{\pi }{4}$]. Figure 2 presents a schematic view for *SC*_*1*_ around *p*_*i,j*_. In this figure, a sample HE is shown with a yellowish oval. Red circles are the boundary points located on the boundary of HE.

For each semi-circular region, we consider several directions shown with green dashed lines in Fig. 2. In fact, the whole angle interval for each semi-circular region is equally divided. Let *D*_*j,i*_ denote the *j*^th^ direction in *SC*_*i*_. Also, ${\varphi }_{j,i}$ denotes the angle made between the x axis and *D*_*j,i*_. Since HE is a region where sharp intensity changes are observed in its boundaries, it is reasonable to verify all the directions in each semi-circular region from this viewpoint. In fact, for all the existing directions in a semi-circular region, it is verified whether or not a significant intensity change is observed along the related directions. In order to do so, firstly we find all the pixels located in direction *D*_*j,i*_. It is possible to consider several discrete values from a pre-defined range denoted by [${r}_{0} {r}_{1}]$ for *R* and find the pixels using the cosine and sine values of ${\varphi }_{j,i}$. Let ${px}_{j,i}^{n}$ denote the pixel located at $({r}_{n},{\varphi }_{j,i})$ polar coordinates. Let *SET*_*j,i*_ denote the set of all pixels which are located on *D*_*j,i*_. Then, for all ${px}_{j,i}^{n}\in {SET}_{j,i}$, we should compute the intensity difference between two consecutive pixels. Let ${df}_{j,i}^{n}$ denote the difference between *n*^th^ and (*n*-1)^th^ pixel located on *D*_*j,i*_. It is computed through the following equation.

1 $$df_{j,i}^{n} = X\left( {px_{j,i}^{n} } \right) - X\left( {px_{j,i}^{{\left( {n - 1} \right)}} } \right)$$

where in (1), $X\left( {px_{j,i}^{n} } \right)$ denotes the intensity value of $px_{j,i}^{n}$.

Then, we verify the values of $df_{j,i}^{n}$ in *D*_*j,i*_ and find $px_{j,i}^{n}$ such that the following conditions are true.

2 $$df_{j,i}^{n} < 0,\;\;\;abs(df_{j,i}^{n} ) > Th_{1}$$

**Figure 3:**
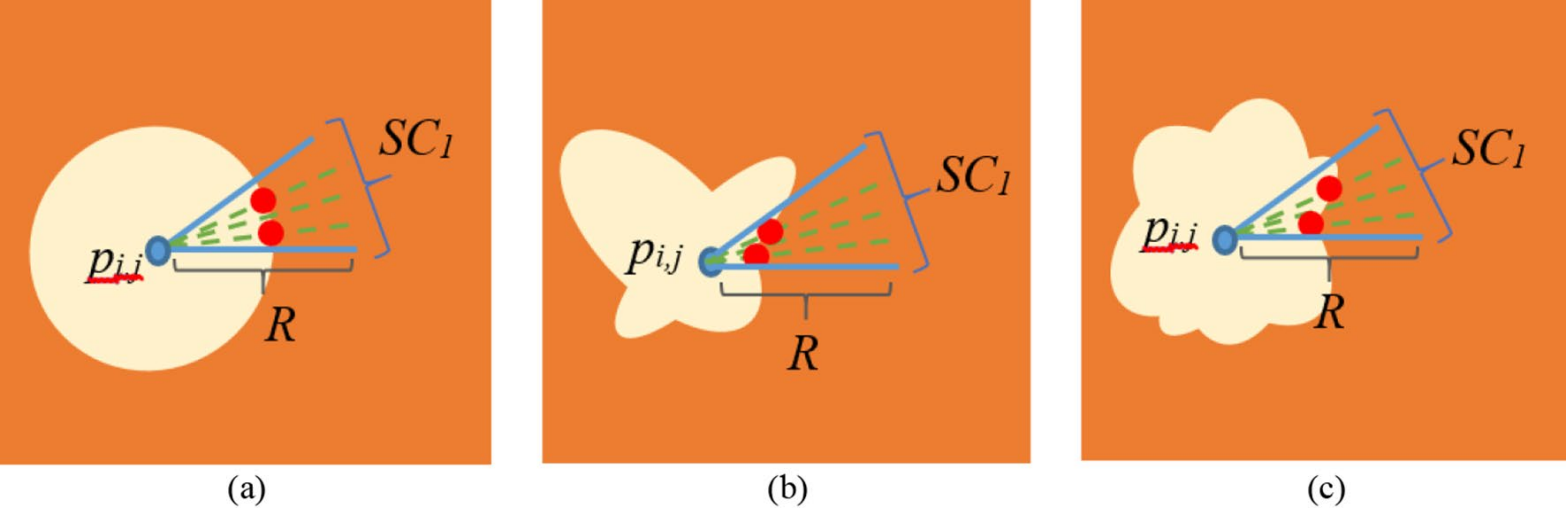
HEs with different shapes.

**Figure 4:**
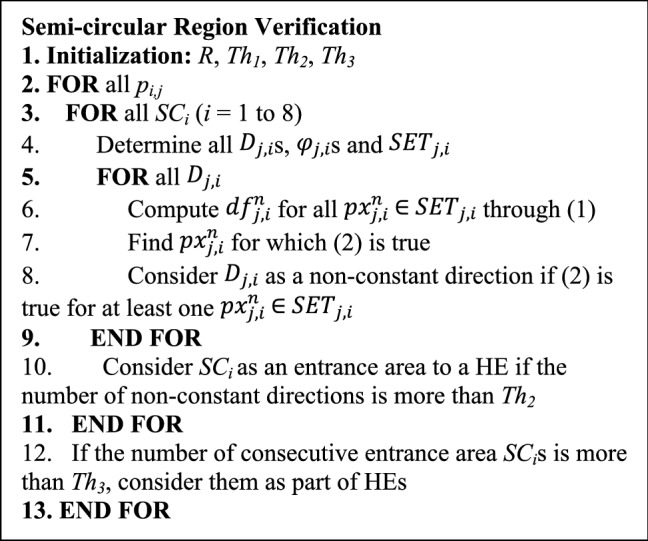
Pseudo-code for semi-circular region verification phase.

It should be noted that *abs*() is the absolute operator and *Th*_*1*_ is a threshold value. If these conditions are true for *D*_*j,i*_, this direction is considered as a non-constant direction. If the number of non-constant directions in *SC*_*i*_ is more than a threshold denoted by (*Th*_*2*_), *SC*_*i*_ is considered as an entrance area to a HE. It should be noted that the pixels which satisfy the above equation, are presented with red circles in Fig. 2. It can be observed that such pixels are not necessarily located at the same distances from the initial pixel, *p*_*i,j*_. Such a point leads to the identification of HEs with different shapes and sizes. Figure 3 presents several samples for HEs with different shapes which can be extracted by the proposed method. For each HE, *SC*_*1*_ is presented and its directions are shown with green dashed lines inside it. As mentioned before, red circles are the boundary points located on the boundary of HE. As obvious in the figure, the boundary points are not necessarily located in the same distance from the initial point, *p*_*i,j*_. The pseudo-code for semi-circular region verification phase is presented in Fig. 4.

### Post-processing

In this phase, optic disc is detected and removed to reduce the number of false positive regions. The processing operations for finding optic disc are explained here.

**Figure 5:**
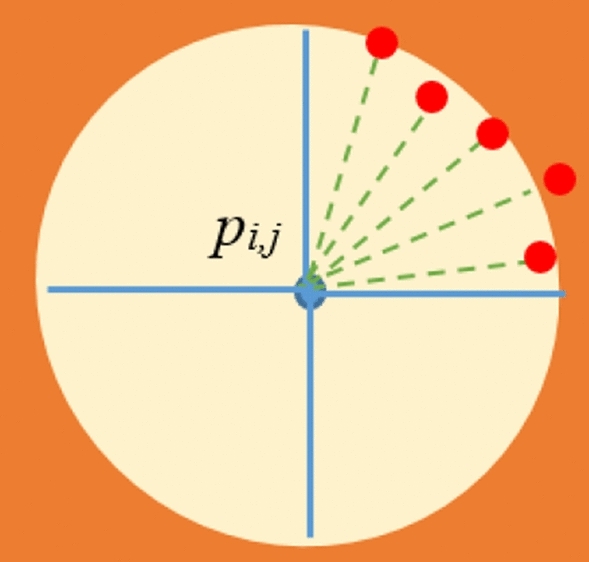
A circle around *p*_*i,j*_ to show the processing operations for optic disc detection.

At first, the noise of image is reduced using a noise reduction method like median filtering. Then, the boundary points are extracted using an edge detection method such as Sobel method. The main idea is to find objects like circles in the fundus image. Initially the dimensions of optic disc are manually computed in several images to specify the dimensions of circles which should be looked for. Next, for each pixel, a circle is considered which is centered at the pixel and the radius is equal to the radius of manually labeled optic disc. Figure 5 presents a circle centered at *p*_*i,j*_. This circle is divided to four quarters. Each quarter is divided into several circle sectors. Along each direction, it is verified whether or not a boundary pixel is found near the pre-determined radius. If the number of directions with such a characteristic is more than a threshold, the related quarter is considered as a candidate quarter. If all the four quarters of circle are candidate, the whole circle is considered as a candidate for optic disc. Since it may be possible to find several circles which are candidates for optic disc, the brightness condition is additionally considered. For each candidate circle, the brightest direction is determined. In fact, the mean intensity of pixels which belong to each direction is computed. For each candidate circle, the direction with maximum mean value is determined. Then, among the candidate circles, the one with the highest mean value is labeled as optic disc.

## Experimental results

In this section the performance of HED-SRA method is evaluated. In order to evaluate the performance of the proposed method, several public datasets are considered. The first one is DIARETDB1^37^ which includes 89 fundus images. 84 images of this dataset include signs of DR while 5 images are completely normal. For capturing these images, a digital fundus camera with 50 degree field of view is utilized. The second one is DIARETDB0^38^ containing 130 images. 110 images have signs of DR while 20 ones are normal. The third one is STARE^39^ which includes 400 retinal fundus images. The fourth one is IDRiD^40^ including 54 images. Finally, the fifth one is SUS-Tech SYSO^41^ consisting of 1219 images.

The performance metrics are sensitivity and specificity. Sensitivity measures the ratio of the number of true positive lesions divided by the sum of the number of true positive lesions and false negative ones. By “specificity”, we measure the ratio of true negative cases divided by the sum of true negatives and false positives. Table 1 presents the values of parameters used in the simulations.

**Table 1:** The values of parameters in the simulations.

Parameter	Value
*Th*_*1*_	0.015
*Th*_*2*_	2
*Th*_*3*_	3
*r*_*0*_	4
*r*_*1*_	25

**Figure 6:**
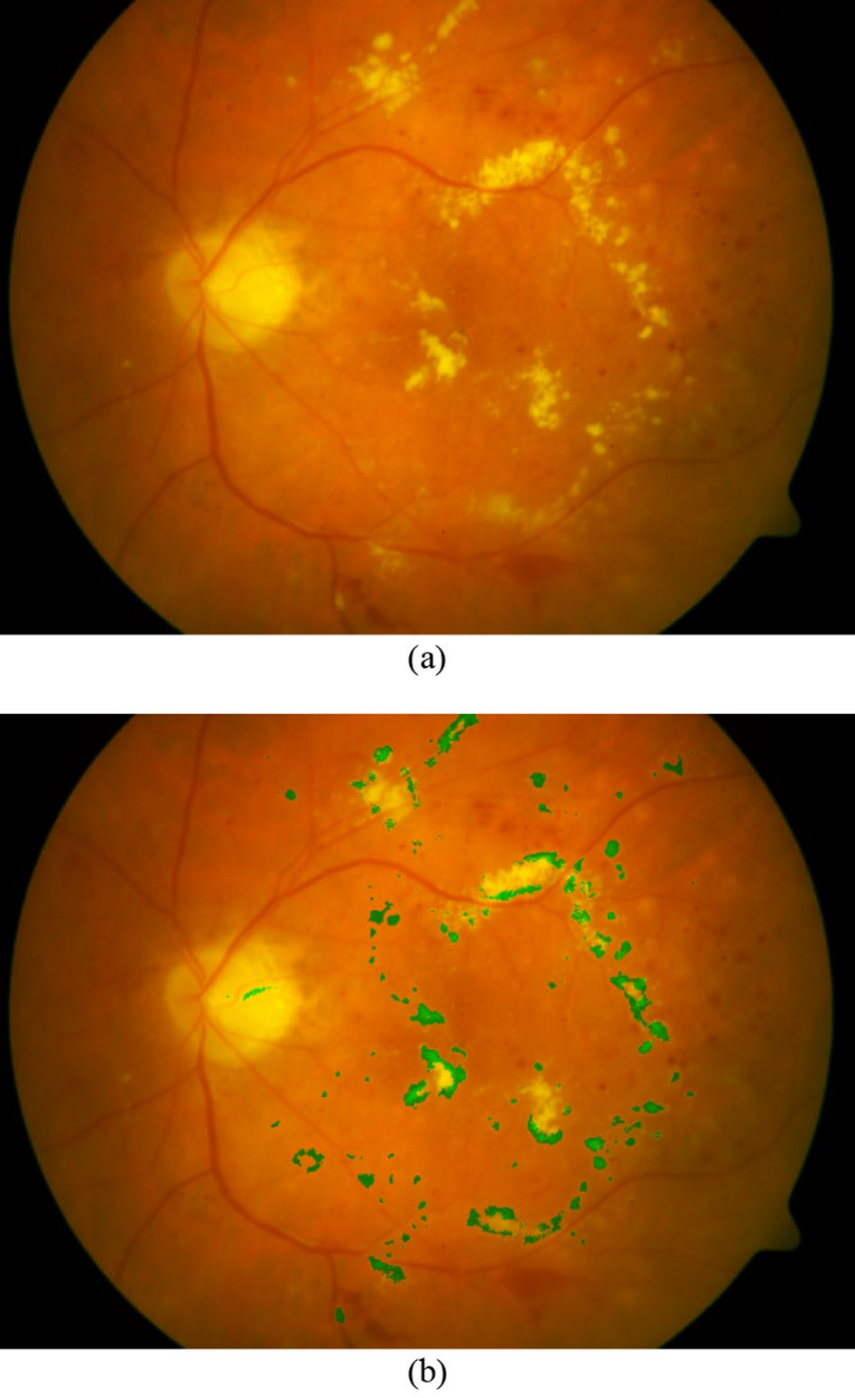
(**a**) A retinal fundus image, (**b**) Extracted HEs by HED-SRA method.

Figure 6 shows a retinal fundus image and its HEs which have been extracted by our HED-SRA method. The extracted HE regions are presented with green color. As can be observed in the figure, almost all the HEs in the image are correctly identified by the proposed method.

**Figure 7:**
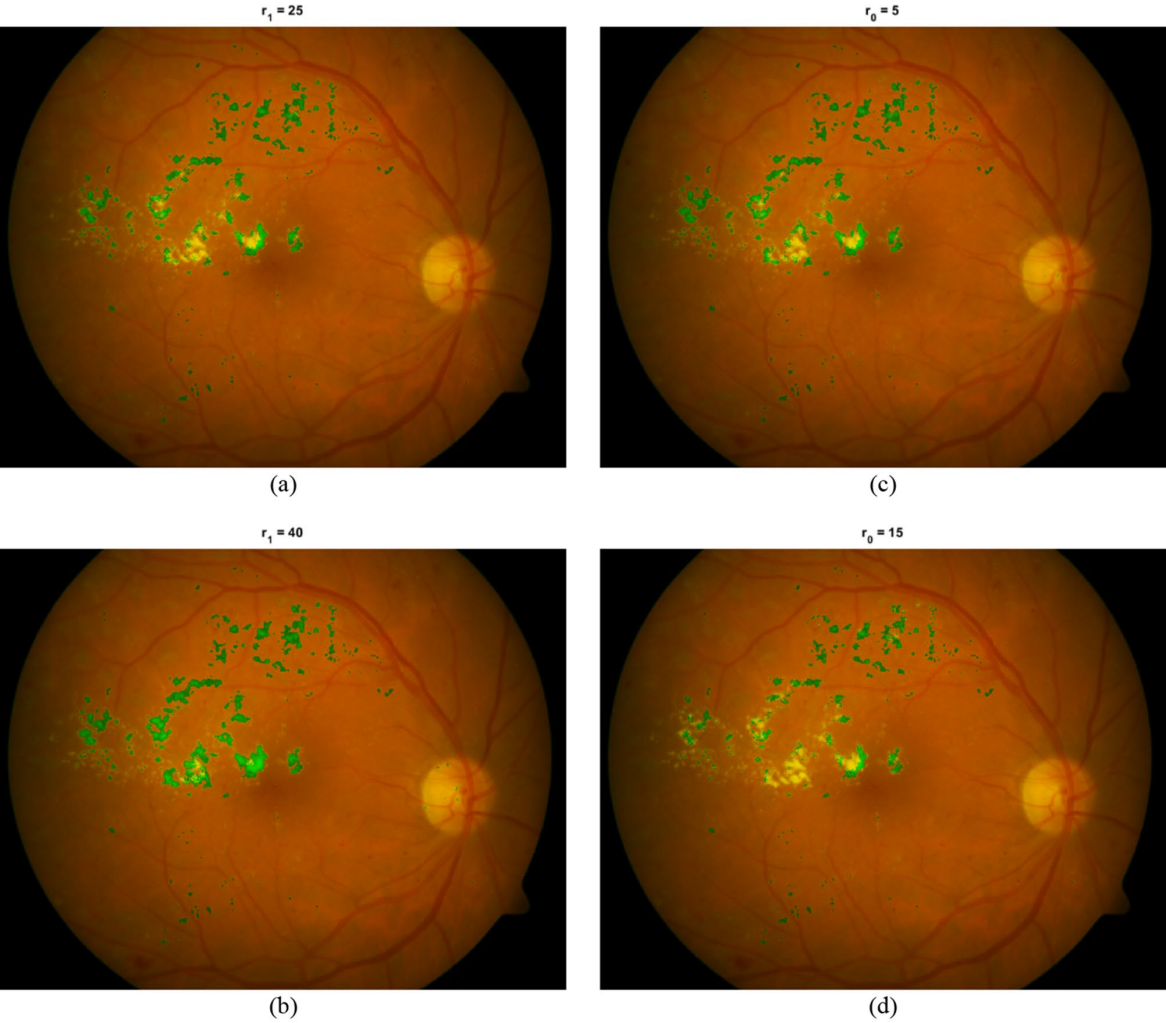
Extracted HEs by HED-SRA for two different values of *r*_*0*_ and *r*_*1*_. (**a**) *r*_*1*_ = 25, (**b**) *r*_*1*_ = 40, (**c**) *r*_*0*_ = 5, (**d**) *r*_*0*_ = 15.

Figure 7 presents the extracted HEs from a retinal fundus image for different values of *r*_*1*_ and *r*_*0*_. Parts a and b show the effect of changing the value of *r*_*1*_. As can be observed, for the higher value of *r*_*1*_, more HEs are extracted by the proposed method. The reason is that by increasing the value of *r*_*1*_, longer distances are searched for the existence of a significant intensity change in semi-circular regions. Therefore, it is more probable to find significant intensity changes and find non-constant directions. Consequently, the conditions for considering a region as a part of HE become simpler and therefore sensitivity also increases. Parts c and d show the effect of change in the value of *r*_*0*_. As obvious in the figures, by increasing the value of *r*_*0*_, the search space for finding significant intensity change becomes more limited and less HEs are extracted.

**Figure 8:**
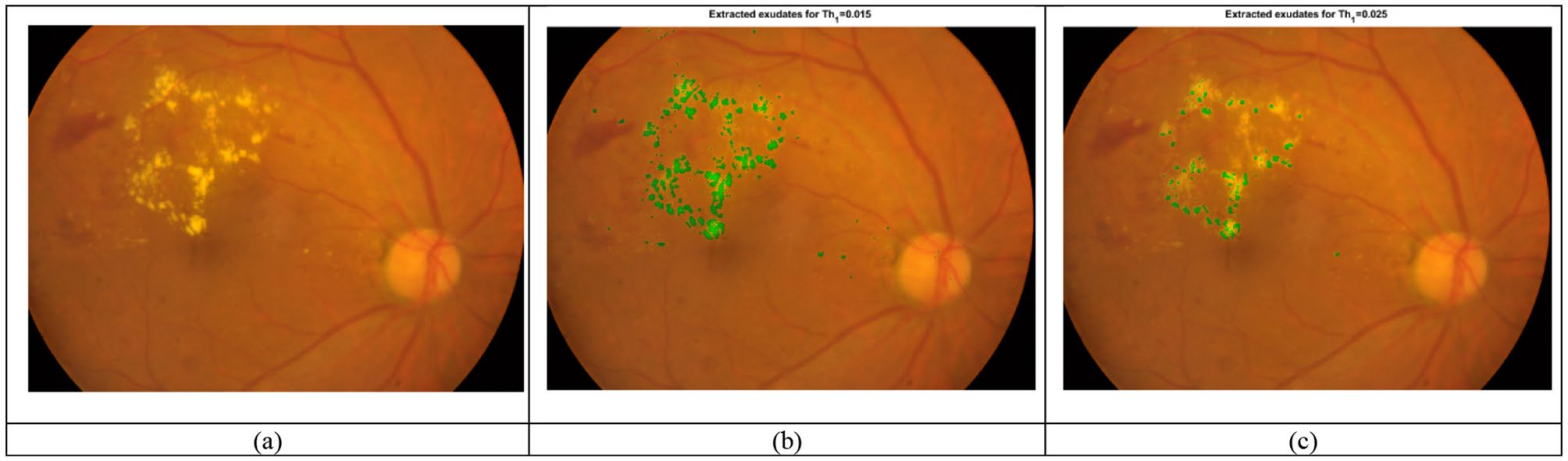
(**a**) A sample fundus image and the extracted exudates by the proposed method for different values of *Th*_*1*_. (**b**) *Th*_*1*_ = 0.015, (**c**) *Th*_*1*_ = 0.025.

In the following, the effect of different values for *Th*_*1*_ is verified. Two different values have been considered for *Th*_*1*_ are considered and the extracted exudates are presented in Fig. 8. As can be observed, for the smaller values of *Th*_*1*_, more exudates have been extracted. In fact, the conditions for considering a region as an exudate become simpler.

**Figure 9:**
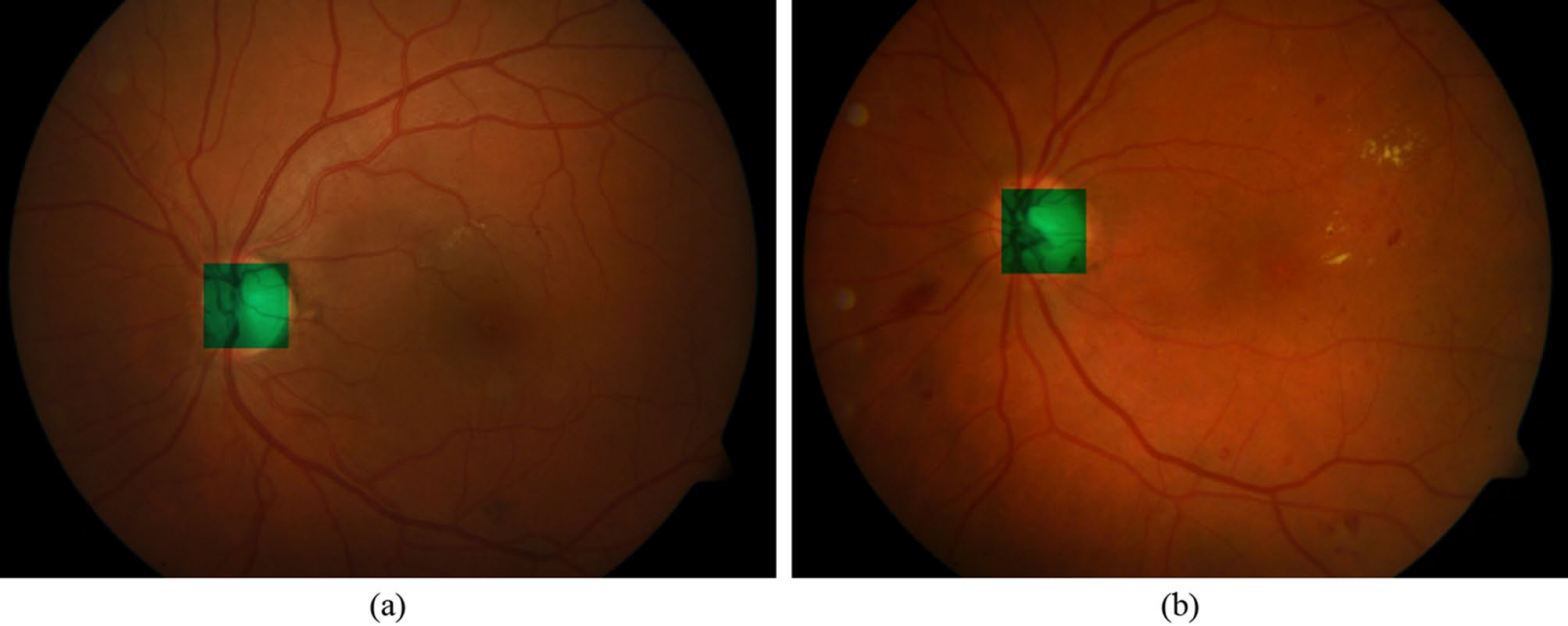
Two sample retinal fundus images and their localized optic disc.

Figure 9 presents two retinal fundus images for which the optic disc localization has been executed. As can be observed, the location of optic disc has been correctly identified in both of images.

In order to compare the performance of the proposed method with the state of the art research works, we have chosen several papers with the same purpose and with the same datasets. For instance, with respect to Diaretdb1, we have selected^9,10,27^ for comparison. Table 2 presents the values of performance metrics for our HED-SRA method and also the methods of^9,10,27,31,42^. For our proposed method, we have also reported the standard deviation. In order to measure the sensitivity and specificity values, we divide the whole image into square patches and measure the intersection between the ground truth and the output image in each patch. If the Intersection over Union (IoU) is more than 0.8, the patch is considered as a correct exudate patch.

**Table 2:** The values of sensitivity and specificity† for different methods on (a) Diaretdb0, (b) Diaretdb1, (c) STARE, (d) SUS-Tech SYSO, (e) IDRiD.

(a)		
Method	Sensitivity	Specificity
HED-SRA	0.92 ± 0.005	0.98 ± 0.004
^31^	0.89	0.99

(b)		
Method	Sensitivity	Specificity
HED-SRA	0.9 ± 0.001	0.98 ± 0.003
^10^	0.81	0.81
^9^	0.88	0.99
^27^	0.91	0.94

(c)		
Method	Sensitivity	Specificity
HED-SRA	0.98 ± 0.002	0.92 ± 0.003
^42^	0.98	0.89

(d)		
Method	Sensitivity	Specificity
HED-SRA	0.95 ± 0.005	0.90 ± 0.011

(e)		
Method	Sensitivity	Specificity
HED-SRA	0.92 ± 0.002	0.95 ± 0.012
^31^	0.815	0.99

^†^Mean ± SD.

In^9^ a deep learning method for segmenting retinal lesions is proposed. Firstly, it is necessary to perform pre-processing operations which include illumination equalization, de-noising, transforming RGB image to CIELAB color space and applying contrast enhancement on the resulting image. There are three main blocks in the neural network architecture. The first block consists of an encoder. Two decoders are located in the second block each of which have their own specific weights. Finally, the third block is a generalization block for binary classification. As obvious in Table 2 the sensitivity value of HED-SRA is slightly higher than^9^ while the specificity values are very close. However, it should be mentioned that in our proposed method, no complicated pre-processing such as illumination equalization is performed. Moreover, there is no need to initial training process.

In^27^ a method for the segmentation of exudates is proposed including three phases. In the first phase, image enhancement is performed using two directional high-pass filtering. Then, anatomical structure such as optic disc and blood vessels are segmented and removed from the image. Finally, exudates are segmented via k-means clustering method which in turn clusters the pixels and updates the centroids. As can be observed in the table, the sensitivity values are close and the specificity of our method is slightly better. Moreover, the initial filtering and the removement of anatomical structures which are executed in^27^ are not necessary in our method.

With respect to Diaretdb0 and IDRiD, we have selected^31^ for comparison. In^31^ a method for automatic diagnosis of exudates is proposed. Firstly, pre-processing operations including optic disc segmentation and blood vessel segmentation are performed which necessitate median filtering, contrast enhancement, and morphological operations. In order to find the best set of features for exudates, different color spaces such as HIS, Lab, Luv, XYZ and RGB are utilized. Then, different statistical features including variance, entropy and standard deviation are used. Finally, fuzzy C-means clustering is used to cluster the pixels. As can be observed, the sensitivity value of HED-SRA is higher than^31^ while specificity values are close. In addition, no operation for optic disc segmentation and blood vessel detection/segmentation are performed in our proposed method.

In order to evaluate the performance of our proposed method on the STARE dataset, we compare it with the method of^42^. In the method of^42^ for exudate identification, firstly optic disc is detected using Fuzzy C-Means (FCM) algorithm. FCM segments the green channel of the image into 7 clusters. In FCM, a membership degree is assigned to each image pixels. The two segments with high intensity are selected from all clusters. Then, inverse surface adaptive thresholding techniques are utilized for the segmentation of exudates. The sensitivity and specificity values for^42^ are equal to 0.98 and 0.89, respectively. Aside from our improved results compared to^42^, it should be noted that the method of^42^ consists of 13 detailed steps and different operations such as Otsu thresholding, and Prewitt edge detection which make it more complicated than HED-SRA method.

## Discussion

**Figure 10:**
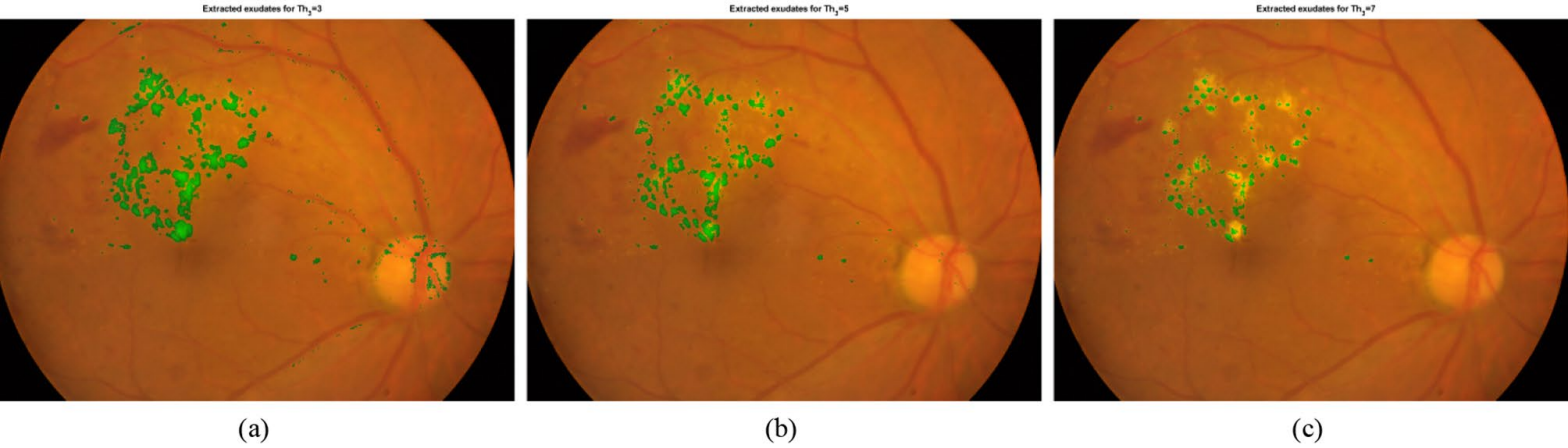
Extracted exudates in a sample fundus image for (**a**) *Th*_*3*_ = *3,* (**b**) *Th*_*3*_ = 5, (**c**) *Th*_*3*_ = 7.

In the following, we present the effect of considering different values for *Th*_*3*_ in the implementation of the proposed algorithm. Figure 10 shows the extracted exudates by our proposed algorithm for three different values of *Th*_*3*_. It can be observed that for the smaller value of *Th*_*3*_, the regions which are falsely detected as exudates become more. In fact, in such conditions, the conditions for considering a region as an exudate become easier. The reason for increasing the false positives is that it may be possible for a pixel to have several semi-circular regions around, however it is not a part of exudate. In fact, the more semi-circular regions with sufficient non-constant directions around a pixel, the more probable the pixel can be considered as a part of HE. However, if the value of *Th*_*3*_ is increased more and reaches to 7, the conditions become difficult and some correct HEs may be missed.

**Figure 11:**
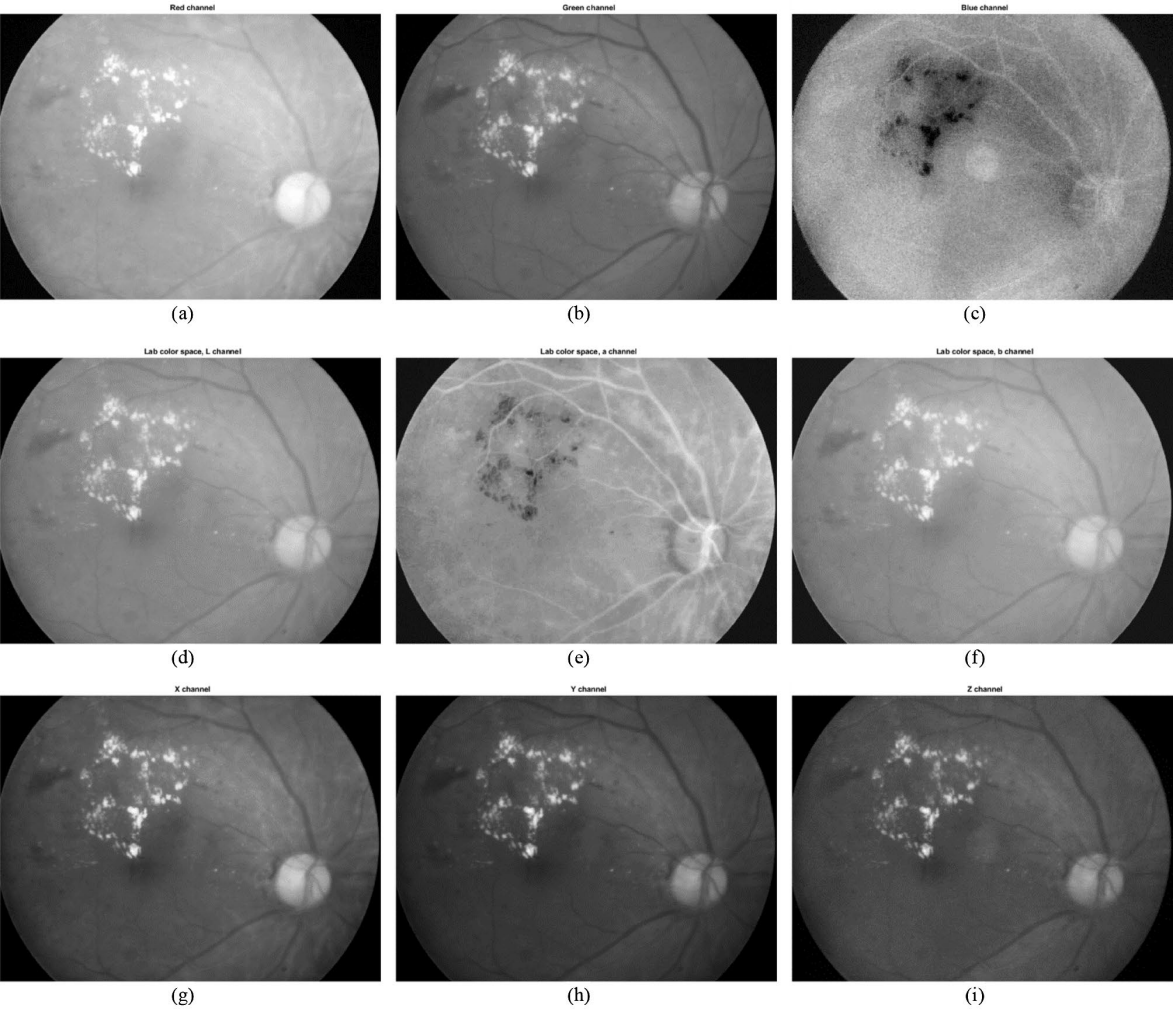
A sample fundus image in (**a**) Red, (**b**) Green, (**c**) Blue channels of RGB color space, (**d**) L, (**e**) a, (**f**) b channels of Lab color space, (**g**) X channel, (**h**) Y channel and (**i**) Z channel.

With respect to different color spaces, we have tested the performance of our proposed algorithm on the images from different color spaces, too. Regarding Lab color space, we firstly present a sample image in rgb and Lab color spaces. In Fig. 11, parts a, b and c show the red, green and blue channels of a sample fundus image, respectively. Also, parts d, e, and f present the L, a, and b channels of the same image in the Lab color space, respectively. In addition, parts g, h, and I show the X, Y, and Z channels of the same image in the XYZ color space.

Since the proposed method is based on the analysis of intensity changes, the contrast between exudates and background is very important. This is the reason why the green channel of RGB color space is chosen for processing. Also, the channels with the highest contrast from all color spaces are selected. With respect to Lab color space, it sounds reasonable to select L channel due to the same reason. Regarding XYZ color space, Y channel is chosen.

**Figure 12:**
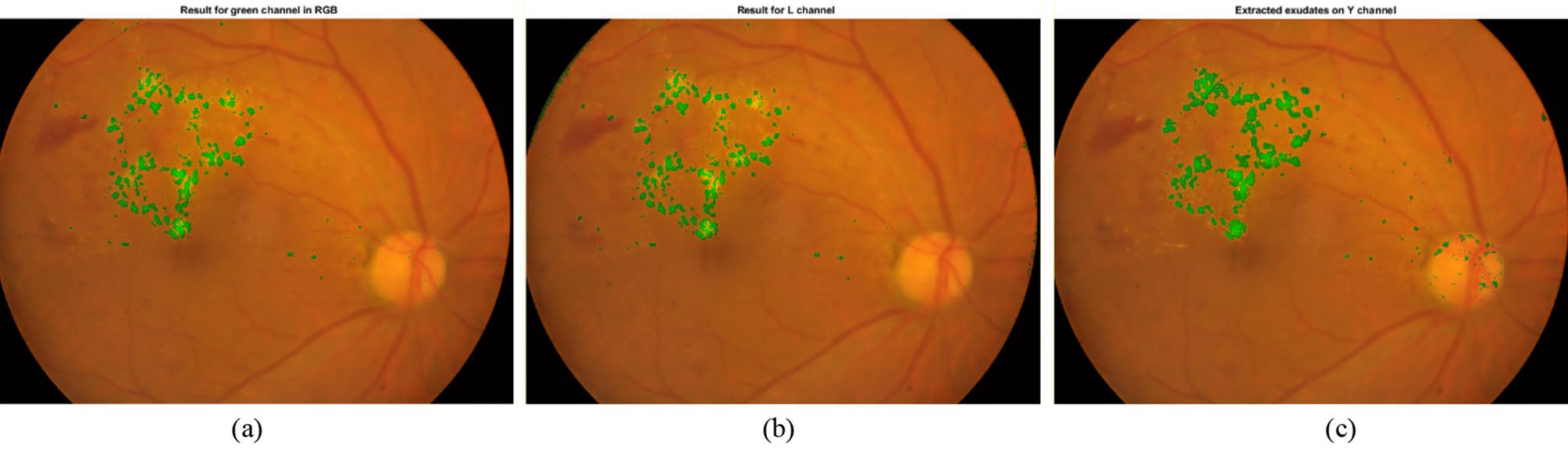
Extracted exudates for (**a**) the green channel of RGB, (**b**) the L channel of Lab and (**c**) the Y channel of XYZ color spaces.

In Fig. 12, the result of our proposed algorithm is presented when applied on G channel in RGB color space, L channel in Lab color space and Y channel in XYZ color space. As can be observed the correct extracted regions using green channel are slightly wider than the L channel. With respect to XYZ color space, the extracted lesions are approximately the same to green channel. However, the small exudates such as the ones located in the left side of the image are better localized in the green channel of RGB color space. Also, the false positive regions extracted are more in xyz color space than rgb.

**Figure 13:**
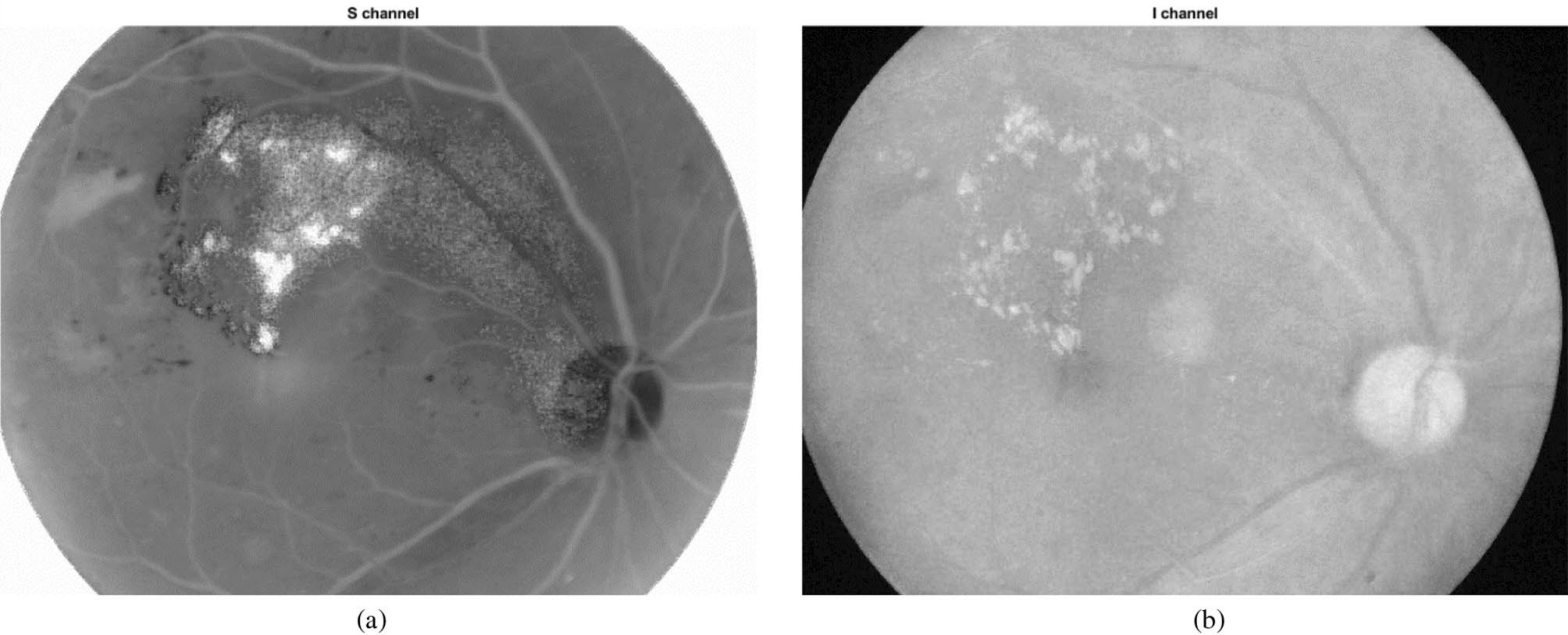
A sample fundus image in (**a**) S channel and I channel of HIS color space.

With respect to HIS color space, in Fig. 13, S and I channels of a sample fundus image are presented.

**Figure 14:**
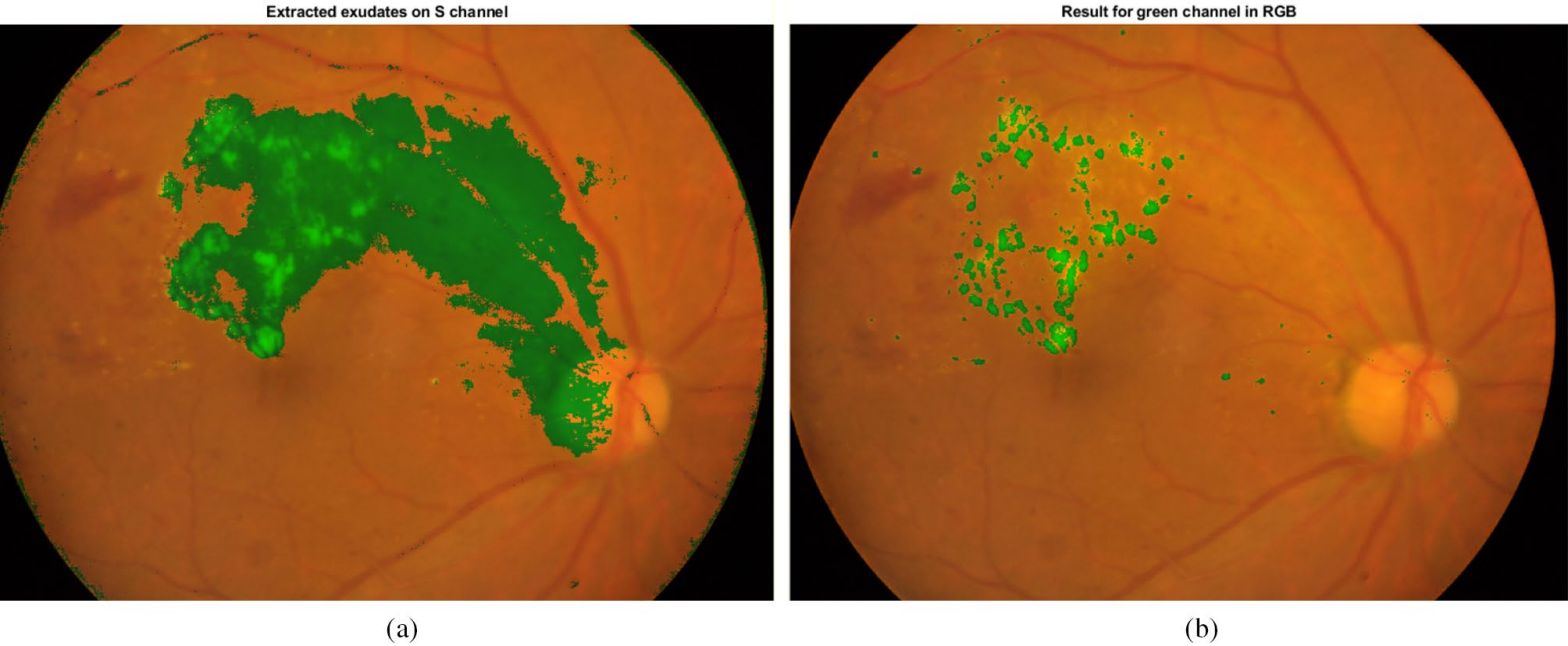
The extracted exudates for S channel of HIS color space and the green channel of RGB color space.

It can be observed that the I channel is very noisy and the contrast of this channel is very low. Therefore, S channel is selected and our proposed method is applied on it. The extracted exudates for S channel of HIS color space and G channel of RGB color space are presented in Fig. 14. As obvious in the figure, the proposed algorithm produces a large number of false positives when applied on S channel. Therefore, working on HIS color space has no advantage and selecting RGB color space is reasonable.

## Conclusions

In this paper, a new method for extracting hard exudates from retinal fundus images is proposed. In the new method, a series of operations are performed on all pixels in the green channel of RGB color space to determine that the region around of them forms a part of HEs. The method works based on following intensity changes in different directions in semi-circular regions. In each semi-circular region, it is verified whether or not a significant intensity change is observed along a certain range in a specified direction. For each semi-circular region, if it is possible to find several directions which include a considerable intensity change, that region is considered as a candidate for HEs. If the number of candidate semi-circular regions is more than a certain threshold, the initial pixel and the candidate circular regions are considered as HEs. It is not necessary to perform contrast enhancement, de-noising or any other complicated pre-processing like blood vessel segmentation in the initial steps. One important advantage of the proposed method is to find HEs with different shapes. In fact, in each direction, a range of distances is followed for finding a significant intensity change. Therefore, it is possible to find a significant intensity change in a close distance from the initial pixel while for the neighbor direction, the significant intensity change is found in a far distance from that. The proposed method is capable of finding HEs with simple computations and high accuracy. The sensitivity and specificity values for our proposed method are 0.92 and 0.9 for Diaretdb0, 0.98 and 0.98 for Diaretdb1, 0.98 and 0.92 for STARE, 0.95 and 0.9 for SUS-Tech SYSO and 0.92 and 0.95 for IDRiD, respectively.

## Data Availability

The datasets generated and/or analyzed during the current study are available in the following links. https://www.it.lut.fi/project/imageret/diaretdb1/. https://www.it.lut.fi/project/imageret/diaretdb0/. https://idrid.grand-challenge.org/Data/. https://cecas.clemson.edu/~ahoover/stare/. https://www.kaggle.com/datasets/mariaherrerot/the-sustechsysu-dataset.
